# The reliability and measurement error of the movement quality assessment for athletes

**DOI:** 10.3389/fspor.2026.1878021

**Published:** 2026-09-09

**Authors:** Gyan A. Wijekulasuriya, Carl T. Woods, Aden Kittel, Paul Larkin

**Affiliations:** 1 Institute for Health and Sport, Victoria University, Melbourne, VIC, Australia; 2 MSA Research Centre, Maribyrnong Sports Academy, Melbourne, VIC, Australia; 3 School of Human Movement and Nutrition Sciences, The University of Queensland, Brisbane, QLD, Australia; 4 Department of Sport and Exercise Science, James Cook University, Townsville, QLD, Australia; 5 Centre for Sport Research with Institute for Physical Activity and Nutrition, School of Exercise and Nutrition Sciences, Deakin University, Melbourne, VIC, Australia

**Keywords:** adolescent athlete, measurement error, movement competency, movement screening, youth athletic development

## Abstract

**Introduction:**

The *Movement Quality Assessment for Athletes* (MQA-A) was recently developed, however, the reliability of its scoring procedure is yet to be determined. Thus, the aim of this study was to determine the intra-rater and inter-rater reliability, agreement between live and video scoring methods and measurement error of the MQA-A.

**Methods:**

The athletic movement quality of 50 adolescent athletes (age: 16.1 ± 0.6 years, mass: 81.7 ± 6.1 kg, height: 173.9 ± 10.5 cm) was measured using the MQA-A by two experienced raters, both live and via video. Intra-rater reliability was determined using the movement quality scores of a single rater, inter-rater reliability was determined for live and video scores and agreement between live and video scoring methods were assessed.

**Results:**

Intra-rater reliability and inter-rater reliability for video and live scoring methods was evident (ICC ≥ 0.90, *K_w_*: 0.39–0.90). There was preliminary evidence for agreement between live and video scoring (ICC = 0.86, *K_w_*: 0.61–0.82).

**Discussion:**

Thus, the MQA-A offers a reliable tool for measuring athletic movement quality in adolescent athletes.

## Introduction

The importance of developing foundational movement skill to support the development of more advanced athletic movements is well espoused across both academia and industry (1–5, 33). In support of this perspective, strength and conditioning coaches have reported the development of foundational athletic movement skills as a key component when preparing adolescents for performance programs (6). Further, an association between the quality of foundational athletic movement and physical fitness has been observed in both adolescent (7–9) and adult cohorts (6). Accordingly, the development of quality athletic movement is a central focus for many youth athletic development programs globally (6). In this context, movement *quality* is defined as the ability to perform a specific task or movement pattern with reference to a pre-determined criteria (10, 11). Although movement quality can be assessed across a range of movement skills (e.g., squat, lunge, push up, running, jumping) (11), the quality of athletic movement is considered foundational for performance across multiple components of physical fitness. Consequently, a range of assessments [Functional Movement Screen, Athletic Ability Assessment (12), Movement Competency Screen] have been developed to evaluate how adolescents perform foundational athletic movements (11).

Athletic movement quality assessments commonly use pre-determined scoring systems and criteria to evaluate the quality of how foundational athletic movements are performed (3, 13). Along these lines, the *Movement Quality Assessment for Athletes* (MQA-A) was developed using an expert consensus approach to establish its components (14). According to these authors, the rationale underpinning its development was to provide a consolidated assessment for practitioners and researchers seeking to assess athletic movement quality within athletic populations. The MQA-A contains six foundational movements: 1) *active straight leg raise*, 2) *the shoulder mobility test*, 3) *squat*, 4) *push up*, 5) *lateral bound*, and 6) *triple hop*. Each movement is scored based on criteria relating to the quality of movement performed, resulting in a composite score derived from the sum of individual movement scores. Of particular interest here is the scoring system, with a 5-point Likert scale anchored around movement quality descriptors verified previously using expert consensus (14). However, our concern is that to date, the scoring process of the MQA-A—and the associated movement descriptors—are yet to be psychometrically evaluated with reference to the inter- and intra-rater reliability, and the agreement between both live and video recorded assessments.

Assessments of athletic movement quality developed for athletic populations typically demonstrate high reliability for composite scores with moderate reliability for individual movement scores (11). Moreover, assessments with high reliability for composite and movement scoring appear to discriminate “good” and “poor” movers (6, 9, 15–18). There has been limited reporting of the discriminant ability of existing athletic movement quality assessments (11, 16). Moreover, low sensitivity to changes in movement quality following exercise interventions have been identified as key limitations of existing athletic movement quality assessments (11, 13). Measurements of error, including the technical error of measurement (TEM), minimal detectable change (MDC) and coefficient of variation (CV), could thus provide important insight the sensitivity of the MQA-A to detect changes in athletic movement quality. To this end, establishing the reliability of the MQA-A is essential to determine its suitability for use in both research and applied practice, while quantifying measurement error could be important for interpretating meaningful changes in athletic movement quality. Therefore, the primary aim of this study was to determine the intra- and inter-rater reliability of the MQA-A, as well as determining the agreement between live and video scoring procedures. A secondary aim was to quantify measurement error and report preliminary normative data for the MQA-A in a cohort of adolescent athletes.

## Materials and methods

### Experimental approach to the problem

A cross-sectional design was employed to examine athletic movement quality using live and video assessments of an adolescent athlete cohort. This cohort was recruited from an Australian secondary sport-specialist high school, with its talent development environment described elsewhere (19, 20). Two raters independently assessed athletic movement quality using the MQA-A under both live and video conditions. Both raters held tertiary qualifications in strength and conditioning, had experience in athletic movement screening (>2 years), and completed specific training in the implementation of the assessment. This training involved jointly reviewing the scoring criteria, establishing cases for each standard of movement, and undertaking a pilot scoring exercise using a cohort of adult athletes prior to data collection. The study was approved by an institutional ethics board (HRE 25-021) and informed consent was sought from parents and/or legal guardians, while adolescent assent was obtained prior to data collection.

### Participants

A sample of 50 adolescent athletes were recruited to participate in this study [Tier 2 (34), Age: 16.1 ± 0.6 y, Height: 173.9 ± 10.5 cm, Body Mass: 81.7 ± 6.1 kg, Table 1]. This sample size was determined *a priori* and aligns with the recommended sample size for reliability studies with two raters (21). Athlete groups recruited were in Years 10–12 in the Australian School Grade system (19, 20) (Table 1, Supplementary Material S1).

**Table 1:** Participant demographic information including age, height, seated height, body mass, maturity offset, sport training age and strength and conditioning training age.

Variable	Overall (*n* = 50)	Male (*n* = 25)	Female (*n* = 25)
Age (y)	16.1 ± 0.6	16.0 ± 0.6	16.2 ± 0.6
Height (cm)	173.9 ± 10.5	180.4 ± 7.1	167.3 ± 9.1
Seated Height (cm)	131.9 ± 4.8	134.6 ± 3.3	129.1 ± 4.5
Body Mass (kg)	81.7 ± 6.1	94.2 ± 7.7	89.1 ± 12.7
Maturity Offset (y)	2.9 ± 0.9	2.6 ± 0.6	3.3 ± 0.9
Sport Training Age (y)	7.3 ± 2.6	7.4 ± 2.8	7.2 ± 2.4
Strength and Conditioning Training Age (y)	3.8 ± 1.3	3.5 ± 1.1	4.1 ± 1.4

To be included in this study, participants were required to be classified as post-peak height velocity (post-PHV), with PHV status, age at peak height velocity and maturity offset being estimated using the methods described by Mirwald et al. (21). Post-PHV was defined as maturity offset greater than 1 year (22). Participants were excluded if they had an injury precluding them from completing the testing procedures.

### Procedures

Participants were instructed to maintain their usual training schedule during the data collection period and to arrive at the testing session in a rested state. Data collection was conducted in the morning to minimise the potential influence of fatigue from daily training sessions on movement performance. All testing was conducted on a wooden basketball court to ensure a consistent testing surface (23).

**Figure 1:**
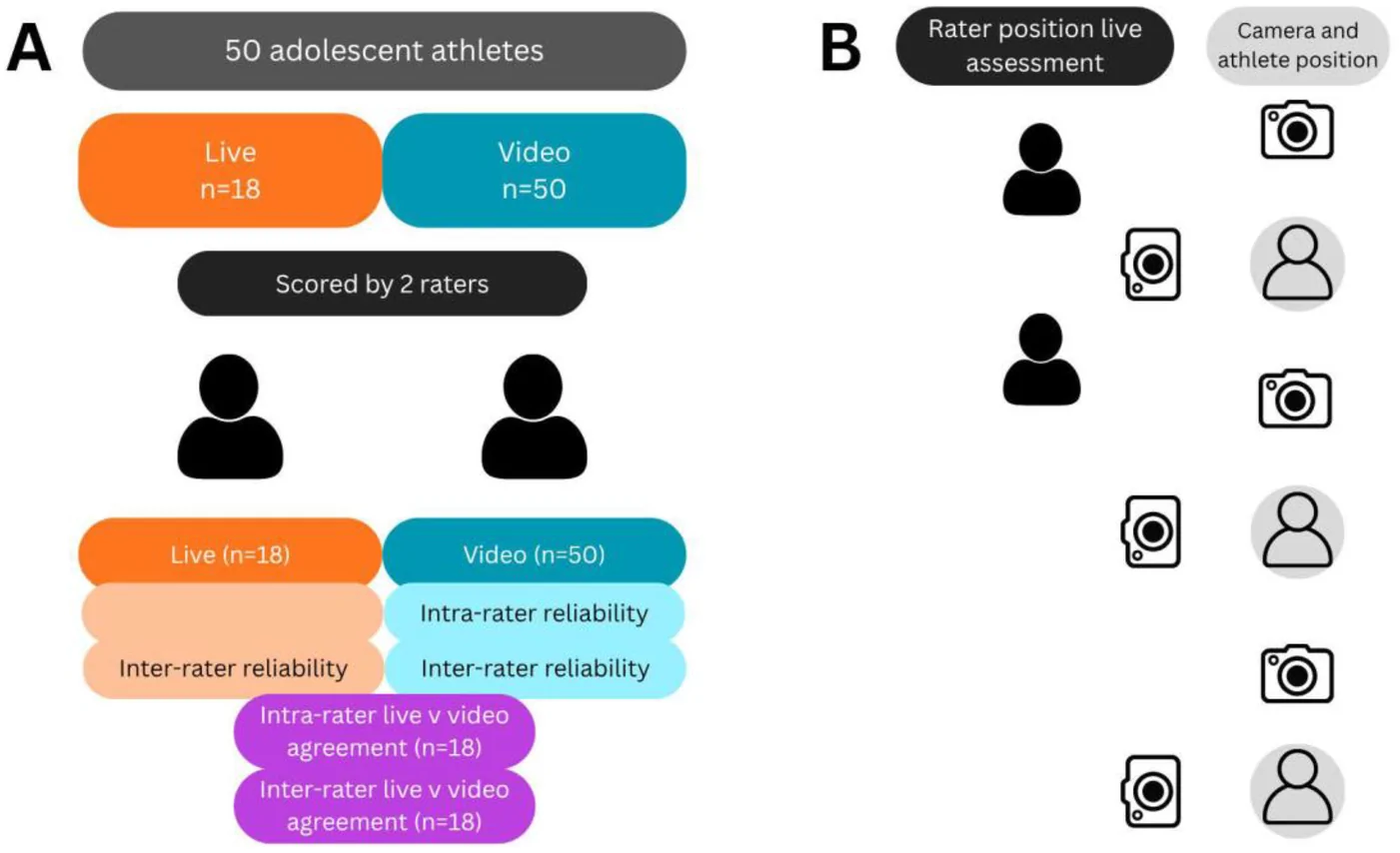
Study design **(A)** and position of camera, participants and raters **(B)** for the determination of live and video reliability of the MQA-A. The “testing zone” in 1B is denoted in grey and live rater positions are denoted by the dark silhouettes.

Athletic movement quality was assessed using the MQA-A (14). Briefly, participants completed the following movements in a standardised order: *active straight leg raise*, *shoulder mobility test*, *squat*, *push up*, *lateral bound* and *triple hop*. Prior to each movement, participants were provided with standardised verbal instructions and viewed a video demonstration of the movement to be performed (14). Thereafter, participants had 5-min to complete a maximum of five practice repetitions before performing the assessed movements. The performance of each movement was recorded using a camera (iPad 10th Gen, Apple, California, USA) positioned on a tripod in the frontal plane, approximately 2-m from a designated “testing zone” (Figure 1). The “testing zone” was denoted by two markers positioned 1.5-m apart. Participants were instructed to perform all movements, apart from the *triple hop*, within this zone. The *triple hop* was initiated from a marker positioned 5-m from the “testing zone”. The two markers were positioned 1.5-m apart within the “testing zone” to assist with the assessment of lateral bound distance (14). For the *squat* and *triple hop*, additional sagittal plane footage was captured using a second camera positioned 1.5-m from the “testing zone”.

### Scoring procedures

Athletic movement quality was assessed for all participants using video scoring, with a random sub-group of 18 participants assessed using live scoring. Following the recommendations from Wijekulasuriya et al. (14), raters positioned themselves either side of the frontal plane camera for movements assessed exclusively in the frontal plane (i.e., *active straight leg raise*, *shoulder mobility test*, *push up*, and *lateral bound*). For the *squat* and t*riple hop*, raters selected their preferred viewing position, either at the frontal plane camera or at the sagittal plane camera (14). In most cases, raters chose to observe these movements from both planes or predominantly from the sagittal plane (see the black silhouettes in Figure 1). A hard copy of the assessment criteria was provided to each rater for reference during scoring. Scores were initially recorded on paper-based data collection sheets and subsequently transcribed to a custom spreadsheet (Microsoft Excel, Microsoft, Redmond, USA).

For video scoring, raters were provided an electronic copy of the assessment criteria (Supplementary Material S2). Video recordings could be reviewed at self-selected speeds, and were able to be paused and replayed as required. Each rater independently scored all movements in a custom spreadsheet (Microsoft Excel, Microsoft, Redmond, USA), being consolidated into one spreadsheet prior to data analysis.

### Intra- and inter-rater reliability

Intra-rater reliability was assessed by having one rater measure the athletic movement quality of all participants via video on two separate occasions. Each assessment occurred 7-days apart. Inter-rater reliability was determined by two raters independently assessing athletic movement quality via video for all participants. Live assessment of athletic movement quality was conducted on a sub-group of 18 participants, whose movement performance were also recorded via video. Data from this sub-group were used to determine inter-rater reliability under live conditions, as well as agreement between live and video scoring methods. Each rater independently scored movement quality under both live and video conditions, with no communication permitted between raters during the assessment process. Upon completion of video scoring, data were uploaded to a shared repository for analysis.

### Statistical analysis

Agreement between raters and scoring methods was assessed using intra-class correlation (ICC) and weighted kappa (*Κ_w_*) statistics. Agreement was determined using ICC for composite scores and *K_w_* for movement scores. The *K_w_* were interpreted according to the guidelines of Landis and Koch (24) (<0.00 = poor, 0.00–0.20 = slight, 0.21–0.40 = fair, 0.41–0.60 = moderate, 0.61–0.80 = substantial, 0.81–1.00 = almost perfect). ICC were interpreted using the recommendations of Koo and Li (25) (<0.50 = poor, 0.50–0.75 = moderate, 0.75–0.90 = good, >0.90 = excellent). Point estimates and 95% confidence intervals (CI) were calculated for the ICC and *Κ_w_* for each agreement calculation. For the *K_w_* point estimates were evaluated in line with the Landis and Koch (24) guidelines whilst the 95% confidence interval was considered when interpreting ICC (25, 35). Inter-rater differences for agreement between live and video scoring methods were determined by comparing the 95% CI of ICC and *Κ_w_* for live and video method agreement for each rater. If the 95% CI did not overlap, the agreement between live and video assessment was considered statistically different between raters. To determine the TEM, standard error was calculated for each agreement analyses using the equation proposed by Hopkins (26). Additionally, MDC was calculated using the equation proposed by Weir (27), while CV (%) for each agreement measure was calculated using the formula proposed by Atkinson and Nevill (28).

To determine preliminary normative statistics, descriptive statistics are reported composite and movement scores. Prior to analysis, the assumption of normality was tested using the Shapiro–Wilk test for composite and movement scores assessed live and via video. These analyses showed composite scores were normally distributed (live and via video) and movement scores were non-normally distributed. Consequently, mean and standard deviation reported for composite scores and median and interquartile ranges were reported for movement scores. Data were tested for differences in athletic movement quality between sexes. Differences in median (movement) were determined using an independent samples median test. Mean differences (composite scores) were calculated using an independent samples *t*-test. All statistical analyses were performed using Statistical Package for Social Sciences (version 30.0.0.0, Chicago, USA) or RStudio (v2025.09.2, packages: “psych”, “vd”).

## Results

### Descriptive statistics

The composite score for athletic movement quality in the observed cohort of adolescent athletes was 27 ± 4 (Table 2). There was no significant difference in composite score between male and female athletes (*p* = 0.273). Female athletes exhibited greater movement quality in the *active straight leg raise* (difference = 1, left: *p* = 0.004) and *shoulder mobility test* (difference = 1, left: *p* = 0.018). There were no other significant differences in movement quality between male and female athletes (*p* = 0.063–0.857).

**Table 2:** Athletic movement quality scores for male and female adolescent athletes (*n* = 50).

Variable	Overall	Male	Female
Active Straight Leg Raise Left (AU)	3 ± 1	3 ± 1	4 ± 2*
Active Straight Leg Raise Right (AU)	3 ± 1	3 ± 1	4 ± 2
Shoulder Mobility Left (AU)	2 ± 2	2 ± 2	3 ± 1*
Shoulder Mobility Right (AU)	3 ± 1	2 ± 2	3 ± 1
Squat (AU)	3 ± 1	2 ± 3	3 ± 3
Push Up (AU)	5 ± 1	5 ± 0	5 ± 2
Lateral Bound (AU)	4 ± 2	4 ± 0	2 ± 2
Triple Hop Left (AU)	2 ± 2	2 ± 0	2 ± 0
Triple Hop Right (AU)	2 ± 0	2 ± 0	2 ± 1
*Composite (AU)*	*27* ± *4*	*26* ± *3*	*28* ± *5*

data are median ± IQR for movement scores and mean ± standard deviation for composite scores (*italics*).

* Indicates movement quality score was significantly greater compared to males.

### Intra-rater reliability—video assessment

Intra-rater reliability ranged from “substantial” to “almost perfect” for movement scores assessed using the video scoring method (*K_w_* = 0.64–0.90, Table 3), while the intra-rater reliability of composite scores was “excellent” (ICC = 0.97, 95% CI: 0.94–0.98). For all movements, the TEM was less than 1 unit and the CV was less than 20% (Table 3). The MDC ranged from 0.6 to 1.7 units for movement scoring by the same rater using the video scoring method (Table 3). Composite score TEM was 1.2 units, CV was 4.8% and MDC was 3.3 units (Table 3).

**Table 3:** Intra-rater reliability for the movement quality assessment for athletes scored by a single rater using the video assessment method.

Movement	*K_w_*	ICC	Intra-rater Reliability Descriptor	TEM (AU)	CV (%)	MDC (AU)
Active Straight Leg Raise Left	0.83 (0.71–0.94)	—	Almost perfect	0.3	10.4	0.8
Active Straight Leg Raise Right	0.64 (0.48–0.81)	—	Substantial	0.2	12.6	0.6
Shoulder Mobility Left	0.72 (0.58–0.87)	—	Substantial	0.4	18.6	1.1
Shoulder Mobility Right	0.78 (0.64–0.92)	—	Substantial	0.4	14.9	1.1
Squat	0.78 (0.64–0.93)	—	Substantial	0.6	17.2	1.7
Push Up	0.90 (0.80–1.00)	—	Almost perfect	0.3	6.6	0.8
Lateral Bound	0.71 (0.52–0.91)	—	Substantial	0.6	19.3	1.7
Triple Hop Left	0.82 (0.62–1.00)	—	Almost perfect	0.2	10.7	0.6
Triple Hop Right	0.77 (0.58–0.97)	—	Substantial	0.3	13.3	0.8
*Composite*	*—*	*0.97 (0.94–0.98)*	*Excellent*	*1*.*2*	*4*.*8*	*3*.*3*

Data are mean values. 95% confidence intervals are reported in brackets for weighted K (*K_w_*) and intraclass correlation coefficients (ICC).

### Inter-rater reliability—video assessment

Inter-rater reliability for movement scoring assessed using video ranged from “fair” to “substantial” (*K_w_* = 0.39–0.74, Table 4), while the inter-rater reliability of composite scoring was “excellent” (ICC = 0.92, 95% CI: 0.94–0.98). The TEM was less than 1 unit for all movements and was 1.9 units for the composite score. The CV for movement scores ranged between 13.5% (*push up*) and 35.0% (*lateral bound*) and the CV for the composite score was 7.5%. Inter-rater MDC ranged between 0.8 and 2.5 units for movement scores (Table 4), while the inter-rater MDC was 5.5 units for the athletic movement quality composite score.

**Table 4:** Inter-rater reliability for the movement quality assessment for athletes scored by two raters using the video assessment method.

Movement	*K_w_*	ICC	Inter-rater Reliability Descriptor	TEM (AU)	CV (%)	MDC (AU)
Active Straight Leg Raise Left	0.54 (0.37–0.72)	—	Moderate	0.6	16.0	1.7
Active Straight Leg Raise Right	0.52 (0.34–0.71)	—	Moderate	0.5	15.2	1.4
Shoulder Mobility Left	0.63 (0.46–0.80)	—	Substantial	0.4	20.1	1.1
Shoulder Mobility Right	0.57 (0.36–0.77)	—	Moderate	0.5	18.5	1.4
Squat	0.68 (0.52–0.85)	—	Substantial	0.8	24.3	2.2
Push Up	0.74 (0.57–0.90)	—	Substantial	0.5	13.5	1.4
Lateral Bound	0.39 (0.20–0.58)	—	Fair	0.9	35.0	2.5
Triple Hop Left	0.70 (0.43–0.97)	—	Substantial	0.3	17.4	0.8
Triple Hop Right	0.70 (0.49–0.91)	—	Substantial	0.4	18.1	1.1
*Composite*	*—*	*0.92 (0.84–0.96)*	*Good to Excellent*	*1*.*9*	*7*.*5*	*5*.*3*

Data are mean values. 95% confidence intervals are reported in brackets for weighted K (*K_w_*) and intraclass correlation coefficients (ICC).

### Inter-rater reliability—live assessment

Inter-rater reliability for the live method of movement quality scoring ranged from “moderate” to “almost perfect” (*K_w_* = 0.48–0.87, Table 5), while “excellent” inter-rater reliability was observed for the composite score (ICC = 0.90, 95% CI = 0.73–0.96). The TEM between raters was a maximum of 0.8 units (*lateral bound*, *triple hop left*) and was 1.5 units for the composite score. The MDC ranged between 0.8 and 2.2 units (Table 5) and was 4.2 units for composite score. The CV between raters was greatest for the *triple hop left* (35.4%) and least for the *active straight leg raise left* (8.3%), while the CV between raters for the composite score was 5.2%.

**Table 5:** Inter-rater reliability for the movement quality assessment for athletes scored by two raters using the live assessment method.

Movement	*K_w_*	ICC	Inter-rater Reliability Descriptor	TEM (AU)	CV (%)	MDC (AU)
Active Straight Leg Raise Left	0.76 (0.53–0.99)	—	Substantial	0.3	8.3	0.8
Active Straight Leg Raise Right	0.63 (0.31–0.95)	—	Substantial	0.4	8.8	1.1
Shoulder Mobility Left	0.74 (0.51–0.97)	—	Substantial	0.3	14.6	0.8
Shoulder Mobility Right	0.48 (0.15–0.80)	—	Moderate	0.5	21.4	1.4
Squat	0.68 (0.49–0.87)	—	Substantial	0.5	13.9	1.4
Push Up	0.87 (0.66–1.00)	—	Almost perfect	0.7	16.1	1.9
Lateral Bound	0.48 (0.23–0.73)	—	Moderate	0.8	26.2	2.2
Triple Hop Left	0.59 (0.24–0.95)	—	Moderate	0.8	35.4	2.2
Triple Hop Right	0.66 (0.35–0.96)	—	Substantial	0.7	29.4	1.9
*Composite*	*—*	*0.90 (0.73–0.96)*	*Moderate to Excellent*	*1*.*5*	*5*.*2*	*4*.*2*

Data are mean values. 95% confidence intervals are reported in brackets for weighted K (*K_w_*) and intraclass correlation coefficients (ICC).

### Live vs. video agreement—intra-rater

There was “substantial” to “almost perfect” intra-rater agreement (*K_w_* = 0.61–0.82) between live and video movement scoring (Table 6). Athletic movement quality score showed “high” intra-rater agreement (ICC = 0.86, 95% CI: 0.56–0.95) between live and video scoring methods, while the intra-rater TEM ranged from 0.3 to 0.6 units for movement scores and was 1.6 units for the athletic movement quality composite score. The CV was between 7.0% (*active straight leg raise right*) and 21.6% (*triple hop left*), while the CV for the composite score was 5.6%. Intra-rater MDC ranged from 0.8 to 1.7 units and was 4.4 for the composite score.

**Table 6:** Intra-rater agreement for a single rater between live and video assessment methods for the movement quality assessment for athletes. .

Movement	*K_w_*	ICC	Intra-rater Reliability Descriptor	TEM (AU)	CV (%)	MDC (AU)
Active Straight Leg Raise Left	0.73 (0.80–1.00)	—	Substantial	0.3	7.3	0.8
Active Straight Leg Raise Right	0.71 (0.60–0.99)	—	Substantial	0.3	7.0	0.8
Shoulder Mobility Left	0.66 (0.53–1.00)	—	Substantial	0.3	14.3	0.8
Shoulder Mobility Right	0.82 (0.47–1.00)	—	Almost perfect	0.3	10.2	0.8
Squat	0.77 (0.36–0.77)	—	Substantial	0.4	11.6	1.1
Push Up	0.77 (0.64–0.90)	—	Substantial	0.6	13.8	1.7
Lateral Bound	0.61 (0.31–0.95)	—	Substantial	0.5	15.2	1.4
Triple Hop Left	0.68 (0.18–0.92)	—	Substantial	0.4	21.6	1.1
Triple Hop Right	0.77 (0.45–0.92)	—	Substantial	0.4	19.5	1.1
*Composite*	*—*	*0.86 (0.77–0.97)*	*Good to Excellent*	*1*.*6*	*5*.*6*	*4*.*4*

Data are mean values. 95% confidence intervals are reported in brackets for weighted K (*K_w_*) and intraclass correlation coefficients (ICC).

### Live vs. video agreement—inter-rater

**Figure 2:**
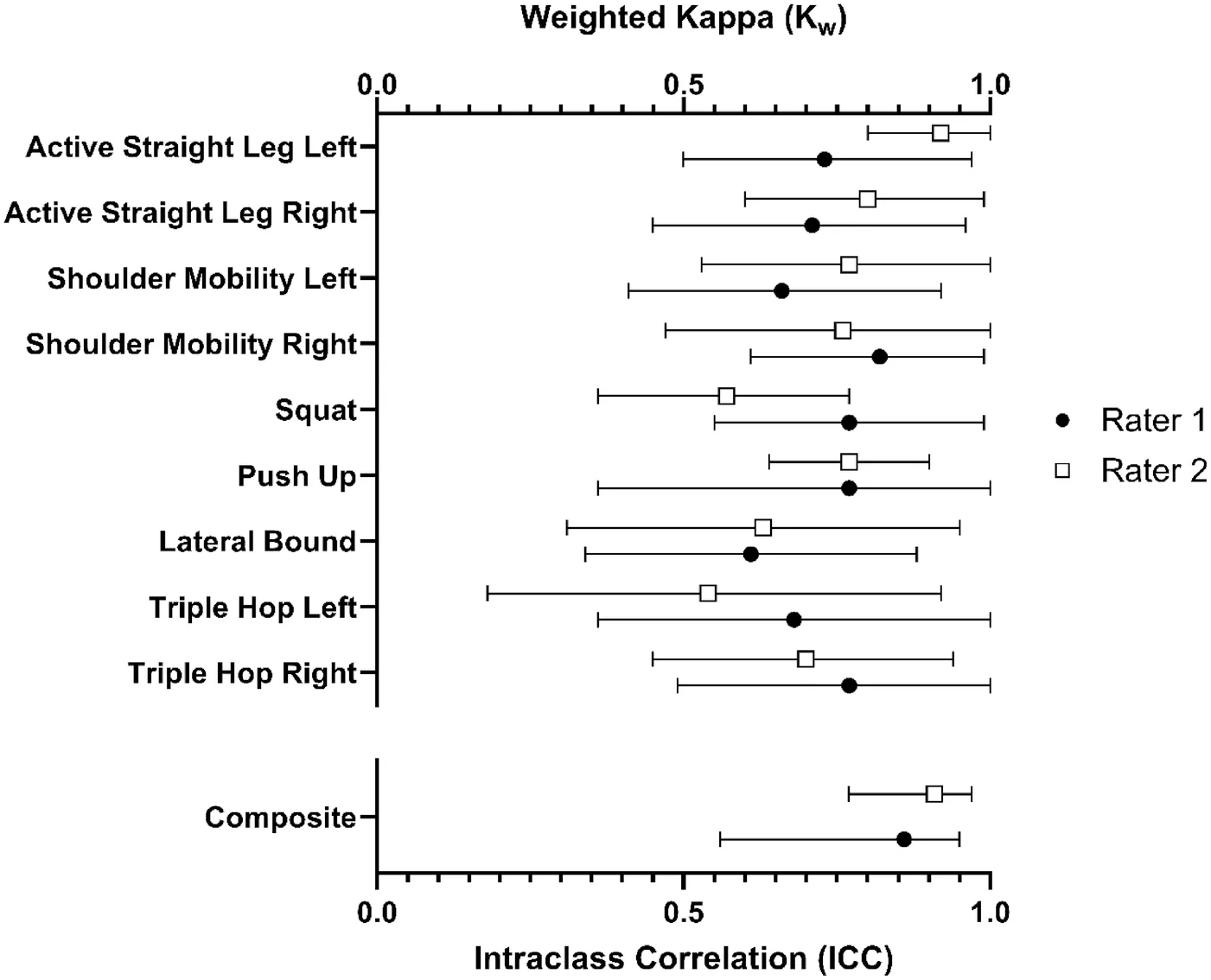
Live and video scoring agreement for two raters using the movement quality assessment for athletes.

Agreement between live and video scoring methods for movement scoring ranged between “substantial” to “almost perfect” (*K_w_* = 0.61–0.82) for rater 1 and “moderate” to “almost perfect” (*K_w_* = 0.54–0.92) for rater 2. Agreement between live and video scoring methods for composite scores was “good” for rater 1 (ICC = 0.86, 95% CI: 0.56–0.95) and “excellent” for rater 2 (ICC = 0.91, 95% CI: 0.77–0.97). The 95% confidence intervals for agreement estimates of both raters overlapped for all movements and composite scores (Figure 2).

## Discussion

The primary aim of this study was to determine the intra- and inter-rater reliability of the MQA-A, as well as the agreement between live and video scoring. The secondary aims were to quantify the TEM, MDC and CV and report preliminary normative data for the MQA-A. Overall, the findings demonstrate that the MQA-A composite score exhibits “high” to “very high” intra- and inter-rater reliability across both scoring methods. The intra- and inter-rater reliability of movement scores ranged from “substantial” to “almost perfect” and “moderate” to “almost perfect” across all video and most live assessments, respectively. Agreement between live and video scoring methods was established, while measurement error was relatively low for both composite and movement scores across all scoring methods and rater combinations. Collectively, these findings support the reliability of the MQA-A measuring athletic movement quality in adolescent athletes.

The MQA-A was found to be reliable for both scoring methods (live and video) and between raters. The intra-rater reliability and inter-rater reliability values are similar to those reported for other athletic movement quality assessments (11, 13). Previous studies have suggested the use of video scoring and measurement of movement quality by a single rater is most likely best practice (10, 11, 16). Practically, video scoring offers several advantages by enabling: i) the opportunity to review movements multiple times, ii) the opportunity to review movements in slower temporal epochs, and iii) recordings to be stored for longitudinal comparisons. Alternately, in youth talent development environments where recording and storage of video is not practical, live scoring may be advantageous. The inter-rater reliability of the MQA-A for live and video scoring could indicate assessment by the same rater is not entirely necessary for this assessment. Consequently, our results suggest that uses of the MQA-A can choose between live, video, single rater and multiple rater methods of assessment, given these methods show acceptable reliability.

The inclusion of TEM, MDC and CV provides important insight to the measurement error associated with the MQA-A. Despite the recognised importance of quantifying measurement error, these metrics are seldom published in studies of athletic movement quality assessments (11). Practitioners and researchers can use the reported TEM and MDC values to establish real differences between groups or changes in movement quality. For example, the data from this study suggest changes of 1 unit for each individual movement and 4 units for composite scores are likely to represent real changes beyond measurement error. Thus, the MQA-A is likely to be sensitive to relatively small but meaningful changes in movement quality. While the feasibility of implementing the MQA-A likely depends on a variety of factors (11, 13, 29), our results provide preliminary evidence for its use with adolescent athletes in talent development environments.

The lack of CV reporting for previous assessments does make it difficult to compare our measurement error with those of others (11). Thus, the TEM statistic offers a standardised alternative for comparison (11). When compared to existing movement assessments, such as the Functional Movement Screen (FMS) (30), the Athletic Ability Assessment (AAA) (12) and the modified AAA (17, 18, 31), the MQA-A appears to demonstrate similar or lower levels of measurement error (11). Within the assessment itself, there were some movements with greater intra-rater and inter-rater variance than others. Namely, the assessment of the *lateral bound* and *triple hop* showed the most variance of the movements assessed in the MQA-A. This likely reflects the increased complexity and dynamic nature of these tasks, which can be a challenge for consistent visual assessment. The use of markerless motion capture to measure joint angles and movement quality could be an innovative solution to improve reliability of athletic movement quality assessment for these, and other movements, in the MQA-A (32). As such, there is impetus for the further redevelopment of athletic movement quality assessments, like the MQA-A, to move towards even more reliable, and feasible, methods of measurement.

While this study provides evidence for the reliability of the MQA-A, it is not without limitations. First, the findings are specific to a cohort of adolescent athletes, and the reliability of the MQA-A in adult populations remains unknown. Second, the sub-group used for the live assessment was relatively small due to constraints in participant availability. As a result, the inter-rater reliability analysis for live assessment and agreement between live and video assessment should be considered preliminary evidence and future research should seek to confirm these findings in larger samples. Third, raters in this study underwent systematic training prior to data collection hence the findings are limited to raters who undergo similar training. The reliability of less experienced or untrained practitioners, for example exercise physiologists or strength and conditioning coaches working with adolescent athletes, is yet to be determined. Practically, the implementation of athletic movement quality assessments within talent development environments will likely be dependent on the resource allocation for strength and conditioning coaches (6, 13). Appreciating this, Wijekulasuriya et al. (14) developed the MQA-A with the aim of being a feasible assessment of athletic movement quality by including “foundational athletic movements” believed to associate with components of physical fitness. Future research may thus seek to establish evidence for or against the association between athletic movement quality, measured using the MQA-A, and components of physical fitness.

## Conclusion

Athletic movement quality can be reliably assessed using the MQA-A. The noted reliability is similar to that which has been established elsewhere (11, 13). The MQA-A demonstrates acceptable reliability across live and video scoring methods, as well as between multiple assessors, providing flexibility for use in research and applied settings. The magnitude of error is comparable to, or lower than, that reported for other movement quality assessments developed for athletic populations, thereby supporting the measurement sensitivity of this assessment. Future work could look to associate scores on the MQA-A with components of physical fitness to further demonstrate its utility in supporting athletic development.

## Practical applications

The MQA-A may be used by practitioners and researchers as a reliable measure for assessing athletic movement quality in adolescent athletes. Applications could include measuring athletic movement quality to i) provide feedback to adolescent athletes about the quality of their foundational movement performance, ii) quantify changes in athletic movement quality across and following an prescribed intervention, or iii) discriminate between athletic movement qualities of different adolescent athlete groups. Collectively, these findings provide preliminary evidence in support of the implementation of the MQA-A in applied settings, with its demonstrated reliability providing a foundation for its use in adolescent athletic populations.

## Data Availability

The raw data supporting the conclusions of this article will be made available by the authors, without undue reservation.

## References

[1] Balyi I Hamilton A. Long-term athlete development: trainability in childhood and adolescence. Olympic Coach. (2004) 16(1):4–9.

[2] Lloyd RS Cronin JB Faigenbaum AD Haff GG Howard R Kraemer WJ. National strength and conditioning association position statement on long-term athletic development. J Strength Cond Res. (2016) 30(6):1491–509. 10.1519/JSC.0000000000001387 26933920

[3] Lloyd RS Faigenbaum AD Stone MH Oliver JL Jeffreys I Moody JA. Position statement on youth resistance training: the 2014 international consensus. Br J Sports Med. (2014) 48(7):498–505. 10.1136/bjsports-2013-092952 24055781

[4] Lloyd RS Oliver JL. The youth physical development model: a new approach to long-term athletic development. Strength Cond J. (2012) 34(3):15–24. 10.1519/SSC.0b013e31825760ea

[5] Wilson G Bird SP O'Connor D Jones J. Resistance Training for Children and Youth: A Position Stand from the Australian Strength and Conditioning Association. Gold Coast (AU): Australian Strength and Conditioning Association (2017).

[6] Burton AM Eisenmann JC Cowburn I Lloyd RS Till K. Developing motor competency in youths: perceptions and practices of strength and conditioning coaches. J Sports Sci. (2021) 39(23):2649–57. 10.1080/02640414.2021.1949189 34225574

[7] Bennett H Fuller J Milanese S Jones S Moore E Chalmers S. Relationship between movement quality and physical performance in elite adolescent Australian football players. J Strength Cond Res. (2022) 36(10):2824–9. 10.1519/JSC.0000000000003903 33651732

[8] Ireton MRE Till K Weaving D Jones B. Differences in the movement skills and physical qualities of elite senior and academy rugby league players. J Strength Cond Res. (2019) 33(5):1328–38. 10.1519/JSC.0000000000002016 28934100

[9] Woods CT McKeown I Keogh J Robertson S. The association between fundamental athletic movements and physical fitness in elite junior Australian footballers. J Sports Sci. (2018) 36(4):445–50. 10.1080/02640414.2017.1313996 28406356

[10] Bennett H Davison K Arnold J Slattery F Martin M Norton K. Multicomponent musculoskeletal movement assessment tools: a systematic review and critical appraisal of their development and applicability to professional practice. J Strength Cond Res. (2017) 31(10):2903–19. 10.1519/JSC.0000000000002058 28614164

[11] Wijekulasuriya GA Woods CT Kittel A Larkin P. The development and content of movement quality assessments in athletic populations: a systematic review and multilevel meta-analysis. Sports Med Open. (2025) 11:7. 10.1186/s40798-025-00813-0 39847263 PMC11757847

[12] McKeown I Taylor-McKeown K Woods C Ball N. Athletic ability assessment: a movement assessment protocol for athletes. Int J Sports Phys Ther. (2014) 9(7):862–73. 25540702 PMC4275191

[13] Pullen BJ Oliver JL Lloyd RS Knight CJ. Assessing athletic motor skill competencies in youths: a narrative review of movement competency screens. Strength Cond J. (2022) 44(4):95–110. 10.1519/SSC.0000000000000673

[14] Wijekulasuriya GA Woods CT Kittel A Larkin P. Establishing expert consensus on athletic movement quality assessment components. J Strength Cond Res. (2026) 40(9):1069–77. 10.1519/JSC.0000000000005478 42190114

[15] Gaudion SL Doma K Sinclair W Banyard HG Woods CT. Identifying the physical fitness, anthropometric and athletic movement qualities discriminant of developmental level in elite junior Australian football: implications for the development of talent. J Strength Cond Res. (2017) 31(7):1830–9. 10.1519/JSC.0000000000001682 27787473

[16] Pearce LA Sinclair WH Leicht AS Woods CT. Physical, anthropometric, and athletic movement qualities discriminate development level in a rugby league talent pathway. J Strength Cond Res. (2018) 32(11):3188–97. 10.1519/JSC.0000000000002350 30540281

[17] Woods CT Banyard HG McKeown I Fransen J Robertson S. Discriminating talent-identified junior Australian footballers using a fundamental gross athletic movement assessment. J Sports Sci Med. (2016) 15(3):548–53. 27803635 PMC4974869

[18] Woods CT Keller BS McKeown I Robertson S. A comparison of athletic movement among talent-identified juniors from different football codes in Australia: implications for talent development. J Strength Cond Res. (2016) 30(9):2440–5. 10.1519/JSC.0000000000001354 26840444

[19] Larkin P Sortino B Carlon T Saunders T Pane C. Gender- and sport-specific normative anthropometric and physical values in talent-identified high school athletes. J Strength Cond Res. (2022) 36(11):3158–66. 10.1519/JSC.0000000000004312 35836319

[20] Larkin P Wijekulasuriya G Greer S. Talent identification of 12-year-old male Australian rules footballers: physical advantages and prognosis for junior and senior national-level selection. PLoS One. (2025) 20:e0317336. 10.1371/journal.pone.0317336 39928704 PMC11809899

[21] Mirwald RL Baxter-Jones ADG Bailey DA Beunen GP. An assessment of maturity from anthropometric measurements. Med Sci Sports Exerc. (2002) 34(4):689–94. 10.1249/00005768-200204000-00020 11932580

[22] Kozieł SM Malina RM. Modified maturity offset prediction equations: validation in independent longitudinal samples of boys and girls. Sports Med. (2018) 48(1):221–36. 10.1007/s40279-017-0750-y PMC5752743 28608181

[23] Tanner R Gore CJ, editors. Physiological Tests for Elite Athletes. 2nd ed. Champaign (IL): Human Kinetics (2012).

[24] Landis JR Koch GG. The measurement of observer agreement for categorical data. Biometrics. (1977) 33(1):159–74. 10.2307/2529310 843571

[25] Koo TK Li MY. A guideline of selecting and reporting intraclass correlation coefficients for reliability research. J Chiropr Med. (2016) 15(2):155–63. 10.1016/j.jcm.2016.02.012 27330520 PMC4913118

[26] Hopkins WG. Measures of reliability in sports medicine and science. Sports Med. (2000) 30(1):1–15. 10.2165/00007256-200030010-00001 10907753

[27] Weir JP. Quantifying test-retest reliability using the intraclass correlation coefficient and the SEM. J Strength Cond Res. (2005) 19(1):231–40. 10.1519/00124278-200502000-00038 15705040

[28] Atkinson G Nevill AM. Statistical methods for assessing measurement error (reliability) in variables relevant to sports medicine. Sports Med. (1998) 26(4):217–38. 10.2165/00007256-199826040-00002 9820922

[29] Rogers SA Hässmén P Alcock A Gilleard WL Warmenhoven JS. Intervention strategies for enhancing movement competencies in youth athletes: a narrative systematic review. Int J Sports Sci Coach. (2020) 15(2):256–72. 10.1177/1747954119900664

[30] Li YM Wang X Chen XP Dai BY. Exploratory factor analysis of the functional movement screen in elite athletes. J Sports Sci. (2015) 33(11):1166–72. 10.1080/02640414.2014.986505 25465117

[31] Woods CT McKeown I Haff GG Robertson S. Comparison of athletic movement between elite junior and senior Australian football players. J Sports Sci. (2016) 34(13):1260–5. 10.1080/02640414.2015.1107185 26525174

[32] Hauenstein JD Huebner A Wagle JP Cobian ER Cummings J Hills C. Reliability of markerless motion capture systems for assessing movement screenings. Orthop J Sports Med. (2024) 12(1):23259671231223789. 10.1177/23259671241234339 PMC10929051 38476162

[33] Lloyd RS Moeskops S Granacher U. Motor skill training for young athletes. In: Lloyd RS Oliver JL, editors. Strength and Conditioning for Young Athletes. Abingdon (UK): Routledge (2019). p. 103–30.

[34] McKay AKA Stellingwerff T Smith ES Martin DT Mujika I Goosey-Tolfrey VL. Defining training and performance caliber: a participant classification framework. Int J Sports Physiol Perform. (2022) 17(2):317–31. 10.1123/ijspp.2021-0451 34965513

[35] Mokkink LB De Vet H Diemeer S Eekhout I. Sample size recommendations for studies on reliability and measurement error: an online application based on simulation studies. Health Serv Outcomes Res Methodol. (2023) 23(3):241–65. 10.1007/s10742-022-00293-9

