# Supplementary material for: The reliability and measurement error of the movement quality assessment for athletes
Source: Front Sports Act Living. 2026 Sep 9;8:1878021. doi: 10.3389/fspor.2026.1878021 (PMC13597740; doi:10.3389/fspor.2026.1878021)
Supplement: Supplementary file 1 [file Table1.docx]

**Supplementary Materials 1** Sport participation and sporting groups of participants

| **Sporting Group** | **Sport** | **Overall** | **Male** | **Female** |
| --- | --- | --- | --- | --- |
| Invasion | Australian Rules Football | 8 | 5 | 3 |
|  | Basketball | 3 | 1 | 2 |
|  | Hockey | 1 | - | 1 |
|  | Netball | 4 | - | 4 |
|  | Football (Soccer) | 2 | - | 2 |
|  | *Total* | *18* | *6* | *12* |
| Striking and Fielding | Baseball | 1 | 1 | - |
|  | Cricket | 2 | 1 | 1 |
|  | Badminton | 1 | 1 | - |
|  | *Total* | *4* | *3* | *1* |
| Net and Wall | Tennis | 2 | 1 | 1 |
|  | Volleyball | 6 | 4 | 2 |
|  | Table Tennis | 1 | - | 1 |
|  | *Total* | *9* | *5* | *4* |
| Physical Performance | Athletics | 3 | 1 | 2 |
|  | Swimming | 5 | 3 | 2 |
|  | *Total* | *8* | *4* | *4* |
| Combat | Judo | 3 | 3 | - |
|  | Taekwondo | 4 | 2 | 2 |
|  | *Total* | *7* | *5* | *2* |
| Target | Golf | 4 | 2 | 2 |
|  | *Total* | *4* | *2* | *2* |
| *Total* |  | *50* | *25* | *25* |
