# Supplementary material for: The reliability and measurement error of the movement quality assessment for athletes
Source: Front Sports Act Living. 2026 Sep 9;8:1878021. doi: 10.3389/fspor.2026.1878021 (PMC13597740; doi:10.3389/fspor.2026.1878021)
Supplement: Supplementary file 2 [file Table2.docx]

**Supplementary Material 2** Assessment criteria for the novel movement quality assessment developed for athletes.

|  | Scoring Criteria | | | | | |
| --- | --- | --- | --- | --- | --- | --- |
| Movement Skill | 0 | 1 | 2 | 3 | 4 | 5 |
| Active Straight Leg Raise | Pain during the movement  Attempting the movement is unsafe due to pain/injury | Bony prominence on ankle (malleolus) below mid-patella | Bony prominence on ankle (malleolus) between mid-patella and mid-thigh  Note, mid-thigh is defined as the halfway point between the anterior sacro-iliac crest and mid-patella | Bony prominence on ankle (malleolus) between mid-thigh and anterior sacro-iliac crest  Note, mid-thigh is defined as the halfway point between the anterior sacro-iliac crest and mid-patella | Bony prominence on ankle (malleolus) in line with anterior sacro-iliac crest  Note, mid-thigh is defined as the halfway point between the anterior sacro-iliac crest and mid-patella | Bony prominence on ankle (malleolus) superior to anterior sacro-iliac crest (between anterior sacro-iliac crest and shoulder) |
| Shoulder Mobility | Attempting the movement is unsafe due to pain/injury | Gap between fists greater than 2 fist lengths  Note, fist length is defined as the distance between midpoint on the distal wrist crease and most proximal point of any knuckle. | Gap between fists greater than a fist length and less than 2 fist lengths  Note, fist length is defined as the distance between midpoint on the distal wrist crease and most proximal point of any knuckle. | Gap between fists less than a fist length  Note, fist length is defined as the distance between midpoint on the distal wrist crease and most proximal point of any knuckle. | Fists touch | Wrists touch |
| Squat | Attempting the movement is unsafe due to pain/injury | Unable to achieve the following criteria:  1. Thigh parallel with ground  2. Upright torso with neutral spine  3. Hip, knee and feet aligned with each other in the frontal plane  Able to achieve:  4. Toes aligned with heels or slight turnout of toes  5. Heels remain grounded | Unable to achieve one of the following criteria:  1. Thigh parallel with ground  2. Upright torso with neutral spine  Able to achieve:  3. Hip, knee and feet aligned with each other in the frontal plane  4. Toes aligned with heels or slight turnout of toes  5. Heels remain grounded | Athlete can achieve all of the following criteria for 5 repetitions of a bodyweight squat:  1. Thigh parallel with ground  2. Upright torso and neutral spine  3. Hip, knee and foot aligned with each other in the frontal plane  4. Toes aligned with heels or slight turnout of toes  5. Heels remain grounded | Athlete can achieve a hips below parallel bodyweight squat for 5 repetitions with all of the following criteria performed consistently:  1. Upright torso and neutral spine  2. Hip, knee and foot aligned with each other in the frontal plane  3. Toes aligned with heels or slight turnout of toes  4. Heels remain grounded | Athlete can achieve a hips below parallel goblet squat loaded at 40% BW or 20kg (whichever is lighter) for 5 repetitions with all of the following criteria performed consistently:  1. Upright torso and neutral spine  2. Hip, knee and foot aligned with each other in the frontal plane  3. Toes aligned with heels or slight turnout of toes  4. Heels remain grounded |
| Push Up | Attempting the movement is unsafe due to pain/injury | Athlete attempts a push up with head, back, hips and heels in a straight line  No complete push up from 5 attempts.  Note, a complete push up is defined as a push up with the upper arm parallel with ground at bottom of movement. | Athlete attempts a push up with:  1. Head, back, hips and heels in a straight line.  2. No excessive elbow flare, shoulders down and away from ears.  No complete push up from 5 attempts.  Note, a complete push up is defined as a push up with the upper arm parallel with ground at bottom of movement. | 1 push up with:  1. No excessive elbow flare, shoulders down and away from ears  2. Head, back, hips and heels in a straight line  3. Upper arm parallel with ground at bottom of movement | 5 push ups with:  1. No excessive elbow flare, shoulders down and away from ears  2. Head, back, hips and heels in a straight line  3. Upper arm parallel with ground at bottom of movement | 10 push ups with:  1. No excessive elbow flare  2. Head, back, hips and heels in a straight line  3. Upper arm parallel with ground at bottom of movement |
| Lateral Bound | Attempting the movement is unsafe due to pain/injury | Unable to perform 6 repetitions in a row | Can achieve 6 repetitions of the lateral bound with evidence of knee valgus and no evidence of hip, knee and foot alignment  Knee valgus defined as the patella closer to the midline of the hip compared to the widest point on the hip  Hip, knee and foot alignment defined as a straight line can be drawn through the hip, knee and heel | 6 repetitions without knee valgus and with hip, knee and foot alignment  Knee valgus defined as the patella closer to the midline of the hip compared to the widest point on the hip  Hip, knee and foot alignment defined as a straight line can be drawn through the hip, knee and heel | 6 repetitions greater than 1.5m without knee valgus and with hip, knee and foot alignment  Knee valgus defined as the patella closer to the midline of the hip compared to the widest point on the hip  Hip, knee and foot alignment defined as a straight line can be drawn through the hip, knee and heel | 6 repetitions greater than 1.5m in 5 seconds with no knee valgus and alignment between hip, knee and foot alignment  Knee valgus defined as the patella closer to the midline of the hip compared to the widest point on the hip  Hip, knee and foot alignment defined as a straight line can be drawn through the hip, knee and heel |
| Triple Hop | Attempting the movement is unsafe due to pain/injury | Inability to complete the triple hop successfully.  Examples of unsuccessful attempts include if an athlete pauses between each hop and if the contralateral foot touches the ground at any point. | One of the following is present during the test:  1. Knee valgus (defined as the patella closer to the midline of the hip compared to the widest point on the hip)  2. Shoulder, hip, knee and heel maligned from the vertical.  3. The athlete does not stick the final hop. | A triple hop performed with  1. No knee valgus (defined as the patella closer to the midline of the hip compared to the widest point on the hip)  2. Shoulder, hip, knee and foot alignment with the vertical. | A triple hop performed with  1. No knee valgus (defined as the patella closer to the midline of the hip compared to the widest point on the hip)  2. Shoulder, hip, knee and foot aligned with the vertical.  3. Heel with no contact on the floor. | A triple hop performed with  1. No knee valgus (defined as the patella closer to the midline of the hip compared to the widest point on the hip)  2. Shoulder, hip, knee and foot aligned with the vertical.  3. Heel with no contact on the floor.  4. Triple extension at toe off during the triple hop |
